# Medical and Common Morality

**Published:** 1890-01-18

**Authors:** 


### The Doctor's Pulpit.

### MEDICAL AND COMMON MORALITY.

The general public look with suspicion on any kind of morality that differs from that which is common and easily understood. If any man suffers from the application of special rules, there is always a tendency to sympathise with the sufferer and to frown upon the special rules and their upholders. The General Medical Council of Great Britain and Ireland is sometimes called upon to exercise discipline against medical offenders; and, on occasion shown, may proceed to strike them off the roll of registered practitioners.

It is of more importance to the public than it is either to the General Medical Council, or to the medical profession as a whole, that such cases should be clearly understood; and it is eminently desirable that the object of the Council in striking any particular name off the rolls should not be defeated by mistaken public sympathy. It is with the intention of serving the public, and not of upholding the interests of the medical profession, that the question of medical morality is here considered in connection with general morality.

It is a not uncommon case for a legally qualified medical practitioner to act as "cover" for an unqualified man; and thus to enable the latter to practise as if he also were "qualified." There is no reason in heaven or earth why any man should not practise medicine or medical electricity, as it is called, until the crack of doom, except—except what? Except the public well-being! But though there is no reason why an ordinary quack doctor should not practise, there is every reason why he should not practise "under false pretences." That is where the whole gist of the business lies. A "quack" doctor has an undisputed legal right to practise physic if he pleases, but he has no right to practise as if he were a "legally qualified practitioner." Moreover, he cannot claim any of the protections extended to legally qualified practitioners; still further, if his want of knowledge results in injury to his patient, he may be proceeded against criminally, with the certain result of adequate punishment in the case of conviction.

Just as a legally "unqualified" person cannot of himself practise as a qualified medical man without incurring the guilt of acting under "false pretences," so neither can a legally qualified man "act as cover" for another in such a way as to make the public believe that the other, or the other's "company," is a "qualified" person or "business concern," without also incurring the guilt and the risk of punishment due to persons acting "under false pretences." It is here that what is erroneously supposed to be medical, and therefore peculiar, morality is shown to be not medical and peculiar, but common and ordinary morality. And it is this common and ordinary morality, or rather "immorality" that the Medical Council have resolved, and very rightly, to root out of the medical profession as far as possible.

The question, therefore, is this: is it ever right to "act under false pretences?" Simple and common morality, which knows nothing of casuistry and special pleading, at once replies that it is never right so to act. But, if it is always wrong, how much more wrong it must be so to act with the mere object of getting money. And if it be wrong so to act with the object of gaining a little money, does not the wrong increase in ever-growing ratio the larger the amount of money gained by the false pretences? A gipsy who tells fortunes for half-a-crown is, on conviction, reprimanded and sent to prison for a month. The promoters of a South Sea Bubble, when their scheme collapses, rouse the execration of a whole nation, and consider themselves fortunate if they escape the clutches of the hangman.

We have no desire to say one angry word against any particular individual. The point to be made and insisted upon is this—that if a medical man deliberately acts "under false pretences" he acts wickedly; and if he enables another person to "act under false pretences" he acts wickedly, and the law says "criminally." His wickedness, supposing it proved, is not medical wickedness, but common wickedness; not a medical crime, but a common crime—the vulgar and base sin and crime of conniving with another man to make the public believe what is not true.

Many people incline to regard the case of a medical culprit solely from the culprit's point of view. They should look at it from the point of view of the families whom, as a registered medical practitioner, the offender may be called upon to treat medically. Is it desirable, is it indispensable that a family medical attendant be a man whose integrity and general character are above suspicion? Let husbands and wives answer this; let fathers and mothers of daughters and sons answer it. If a man has proved himself so destitute of honour as to connive with another to "act under false pretences" for money, is it likely that he will be proof against other temptations to which his inclinations may especially expose him? He who has once transgressed against the code of plain truth and honesty for the very poor object of getting money, will almost inevitably transgress against other parts of the code of virtue if the temptation be sufficiently strong. He has proved his disregard for strict truth and honourable conduct. Who can trust him again?

It may be said, why is the qualified medical man punished and his unqualified fellow-scoundrel allowed to escape? For the simple reason that the unqualified person is not a medical man. It may also be asked whether or not, in the event of being struck off the rolls, the offender can still continue practising? Certainly he can. But he cannot now practise as a registered medical practitioner. There is no question of overpunishment, or of vindictiveness. The Medical Council have decided, and the law has more than once upheld the decision, that such a person has disgraced the medical uniform, and therefore they have decreed that he shall no longer wear that uniform. Whoever believes in the elementary virtues of mere truth and honesty, and whoever desires to be able to trust his family doctor with the honour of his household, will thank the Medical Council in such cases for doing a plain duty in plain and unyielding fashion. So far as we are aware, the Council has never yet shown itself either narrow minded or unjust.

A considerable acquaintance with facts bearing upon such cases as are here considered shows that there is much laxity of moral opinion among both the public and a certain order of medical men, on the point of "covering" unqualified medical practitioners. It is often argued that there are men destitute of legal qualifications whose practical skill and experience are superior to those of many medical men having registered qualifications. That is probably quite true in a certain number of cases. It is painfully evident that no degrees, however high, can always ensure practical capacity. But when all this is allowed, it does not follow that the skilful, but legally unqualified, man is entitled to cheat the public into a belief that he is legally qualified. If his skill be really so great, let him rely upon it for his business success. The public has always a warm side to capable men who are under the ban of professional ostracism. A man who practises medicine or surgery without legal qualifications is not thereby necessarily doing any moral wrong. Why should he turn himself from a possibly honest man into a certain cheat for the sake of gaining respectability or money? Let him accept the situation frankly, and face the world on his plain and actual merits. The public cannot hold itself guiltless of aiding and abetting dishonesty, knavery, and criminality when it makes it profitable for any man to pretend to be what he is not. Those who take the opposite view from this, and regard the matter as of no practical importance, have failed to comprehend the very first principles of elementary morality.

